# RNA silencing in plants: Flash report!

**DOI:** 10.1186/1758-907X-1-13

**Published:** 2010-06-30

**Authors:** Rebecca A Mosher, Mathew G Lewsey, Padubidri V Shivaprasad

**Affiliations:** 1 Department of Plant Sciences, University of Cambridge, Cambridge, CB2 3EA, UK; 2 Plant Biology Laboratory, The Salk Institute for Biological Studies, La Jolla, CA 92037, USA

## Abstract

Earlier this year plant scientists met in Santa Fe, New Mexico at the Keystone Symposium "RNA Silencing Mechanisms in Plants". Sessions included small RNA biogenesis and signalling, development and stress responses, small RNA-directed DNA methylation, and interaction with pathogens. This report highlights some of the prominent and recurring themes at the meeting and emerging arenas of future research.

## Introduction

Many of the seminal discoveries in RNA silencing were made in plant systems and, due in large part to excellent genetics, plant science continues to contribute greatly to our understanding of small RNA biology. At the Keystone Symposium "RNA Silencing Mechanisms in Plants" in Santa Fe, New Mexico, over 150 plant scientists met to share their latest research. Many of the topics discussed will undoubtedly increase understanding of RNA silencing in diverse systems.

## The great migration

Movement of small RNAs over both long and short distances was a major topic at the meeting. The systemic aspect of RNA silencing is well documented [1,2], yet the nature of the systemic signal and the biological significance of this movement are unknown. Marja Timmermans (Cold Spring Harbor Laboratory, USA) presented evidence that diffusion of tasi-ARFs from their point of synthesis at the adaxial leaf surface generates a gradient of *ARF3 *repression and restricts ARF3 activity to the abaxial domain[3]. The tasi-ARF gradient combined with abaxial *ARF3 *expression establishes a discreet domain of ARF3 accumulation, helping define leaf polarity.

To investigate whether cell-to-cell mobility is a general feature of small RNAs, Olivier Voinnet (Institut de Biologie Moleculaire des Plantes, France) bombarded GFP siRNAs into *Arabidopsis *leaves that constitutively expressed GFP and detected patches of silencing spreading from the bombarded cells [4]. To rule out movement of silencing intermediates generated from the target transcript, fluorescently-labelled siRNAs were also bombarded and shown to spread into neighbouring cells. Interestingly, when bombarded near a vein, siRNAs entered the vasculature system and moved long distances. To investigate the potential for long-range movement of siRNAs, the Voinnet group isolated a T-DNA mutant at the siRNA-generating inverted repeat *IR71*. This mutant lacks siRNAs from a portion of the inverted repeat, yet when a wild-type scion was grafted onto mutant rootstock, siRNAs were detected in roots from the entire inverted repeat, demonstrating that endogenous siRNAs or an siRNA precursor transcript from this locus move from shoot to root [5].

David Baulcombe (University of Cambridge, UK) also described analysis of *Arabidopsis *grafts to demonstrate that small RNAs are capable of long distance travel. Using deep sequencing, long-distance movement of siRNAs from thousands of genomic loci was identified [6]. Grafting of wild-type scions onto *dcl2 dcl3 dcl4 *triple mutant rootstocks eliminated the possibility that longer transcripts might transit before being cleaved into siRNAs in the distal tissue. These analyses indicate that siRNAs from some loci are mobile, while other siRNAs are not, and that movement of siRNAs might be responsible for many of the siRNAs that accumulate in root tissue. The Voinnet and Baulcombe groups demonstrate that mobile siRNAs are functionally indistinguishable from locally synthesized siRNAs in that they target DNA methylation in the cells that receive the signal [5,6].

There is evidence that the plant vasculature system is a highway of small RNA molecules. Julia Kehr (Campus Montegancedo Universidad Politecnica, Spain) addressed the biological relevance of this transport route by deep sequencing the small RNA population in *Brassica napus *phloem sap under different nutrient conditions. Several new sRNAs were identified that change abundance in phloem sap independently from the levels in leaves or roots, and it appears clear that movement of many sRNAs is controlled in response to nutrient conditions. For at least one molecule (miR395a), movement of the mature miRNA seems to enhance expression of the precursor in distal tissue [7]. These results offer exciting clues that small RNAs in the phloem might signal changes in environmental conditions.

## The sins of the fathers

A silenced state might be transmitted from parent to progeny [1,8] in experimental systems but it is not known whether this phenomenon involves endogenous loci and or whether it has biological significance. A clue to the answers was presented by Olivier Voinnet, who demonstrated that the large inverted repeat *IR71 *is transcribed in response to stress. Small RNAs from *IR71 *cause DNA methylation in *cis *and are able to silence reporter transgenes in *trans *[5], indicating they might generate stable epigenetic marks in response to stress. The next challenge is to find out whether mRNAs or protein coding genes are targeted by siRNAs from *IR71 *or other inverted repeats.

Jerzy Paszkowski (University of Geneva, Switzerland) also described how the environment might affect silencing by suppression rather than activation of silencing-related RNAs. He presented evidence that transcriptional gene silencing in *Arabidopsis *is released after a shift from low temperature to elevated temperature. Silencing was re-established when plants were returned to normal growth conditions, but specific genomic regions remained de-repressed. Further analysis indicates that one such genomic region contains a *Copia*-like retrotransposon now known as *Onsen *(Japanese for "hot spring"). Although *Onsen *remained transcriptionally active after heat treatment, no new insertions of the element were detected in wild-type plants, either somatically or in the germline. Transposition of *Onsen *was detected in the *nrpd1 *(RNA Pol IV) mutant, but only in the generation following the heat stress of seedlings. Paszkowski speculates that a variety of transposable elements in the genome respond to distinct environmental stimuli and under appropriate conditions could transpose to increase genetic diversity in progeny.

Post-transcriptional repression of transposable elements such as *Onsen *by Pol IV-dependent siRNAs and a delay in the timing of transposition would buffer the genome against transient environmental fluctuations. A similar process, described by Janne Lempe (University of Washington, USA), buffers genetic and environmental variation in order to maintain robust phenotypes. This variation is unmasked in the absence of functional HSP90 [9] and in the small RNA mutants *nrpe1 *and *nrpd2/e2*. Notably *nrpe1 *(RNA Pol V) mutants of *Arabidopsis *show significantly increased phenotypic variation and uncover cryptic genetic variation.

Another condition known to trigger genetic and epigenetic alterations in the genome is the blending of variant genomes through interspecific hybridization. David Baulcombe is also investigating how small RNA populations change during hybridization between cultivated and wild varieties of tomato. Analysis of small RNA populations in a series of introgression lines indicates that hybridization activates small RNA production from specific genomic regions. This alteration in epigenetic state is reflected in altered gene expression, creating transgressive (beyond the parental range) expression states in introgressed lines. Surprisingly, siRNA activation is not immediate upon hybridization, indicating that critical epigenetic control might occur in the germline, similar to the retrotransposition of *Onsen*. These tantalizing results indicate that RNA silencing and transgenerational effects will be a fertile field for future research in both plants and animals.

## Two's company; three's a crowd

Another recurring theme at this meeting was the complex interactions between different polymerases. Through duplications of Pol II, plants have evolved two additional DNA-dependent RNA polymerases (Pol IV and Pol V). Pol IV and Pol V contain distinct largest subunits (encoded in *Arabidopsis *by *NRPD1 *and *NRPE1*, respectively) and are specialized for siRNA production and transcriptional gene silencing [10]. Genetic evidence suggests that Pol IV generates a precursor of small RNA production but, until now, such transcripts have eluded detection. Craig Pikaard (University of Indiana Bloomington, USA) offered the first biochemical evidence of Pol IV activity by demonstrating elongation of "paused transcription" templates.

If Pol IV is limited to "paused transcription" templates *in vivo*, another polymerase might be required for initiation of small RNA synthesis. Xuemei Chen (University of California Riverside, USA) described a weak allele in the second largest subunit of Pol II, *NRPB2*, which indicates that Pol II might transcribe a subset of loci regulated by Pol IV [11]. In addition to pleiotropic developmental defects expected from a Pol II lesion, this mutation resulted in reduced siRNA accumulation and derepression of a set of intergenic low-copy-number repeat sequences. Further analysis determined that Pol II is present at all siRNA loci tested and increases Pol IV occupancy at a subset of these loci. Pol II also recruits Pol V to some loci by generating a scaffold transcript initiating outside the region of small RNA production. Thus, noncoding transcription by Pol II plays a central role in coordinating actions of the other two polymerases. These studies show that Pol II, Pol IV, and Pol V have non-redundant roles in siRNA production, thus revealing the complexity of siRNA metabolism.

Polymerase functions are even more intricate in maize, where there are three genes for the second largest subunit of Pol IV. Jay Hollick (University of California Berkeley, USA) described how these paralogues have subfunctionalized. Loss of one gene, *Rmr7*, causes almost complete lack of 24 nt siRNAs but does not cause the developmental phenotypes observed when all Pol IV activity is lost through mutation of the largest Pol IV subunit, *Rmr6 *[12]. The complexity of polymerase interactions was highlighted by experiments demonstrating competition between Pol IV and Pol II for DNA binding sites in maize. Like *rmr6 *mutants, maize plants with mutations in *Rmr1*, encoding a protein with similarity to SWI/SNF nucleosome remodeling enzymes, lack 24 nt siRNAs. However transposable elements are reactivated only in *rmr6 *plants, indicating that repression of transposable elements is due to exclusion of Pol II from the chromatin by Pol IV rather than siRNA production and RNA-directed DNA methylation [13]. Our understanding of polymerase interactions will undoubtedly advance as genetic and genomic resources improve in maize and other grasses.

## An ancient battle

During replication viruses produce double-stranded RNA that is recognized and cleaved by host Dicers. These primary siRNAs also combat the virus by "slicing" single-stranded viral transcripts, thereby triggering secondary siRNA formation via host RNA-dependent RNA polymerases (RDRs). RDR activity is important in excluding potato virus X from the meristem of *Nicotiana benthamiana *- an important aspect of defence against this virus [14]. Shou-Wei Ding (University of California Riverside, USA) described how *Arabidopsis *RDR1 and RDR6 are required for potent defence against cucumber mosaic virus (CMV) [15]. During CMV infections, the virus-encoded 2b silencing suppressor protein greatly reduces the production of viral secondary siRNAs, most likely by inhibiting RDR6 activity. Potentially a second CMV component acts co-ordinately to suppress RDR1. Since RDRs are also involved in regulation of endogenous plant signalling processes [16], it remains to be investigated whether repression of these RDRs by CMV might also have indirect effects on the host.

Early evidence that viruses might directly manipulate host plant processes *via *RNA silencing was presented by Mathew Lewsey (University of Cambridge, UK). He demonstrated that the CMV 2b protein repressed responses to the phytohormone jasmonic acid (JA) [17]. JA does not regulate anti-viral defence, but it does inhibit herbivorous insects, such as the aphids known to transmit CMV [18]. Therefore CMV suppression of JA signalling might enhance spread of the virus. This silencing suppressor might alter hormone signalling through inhibition of RDR activity and subsequent secondary siRNAs production, or by disruption of AGO1 or AGO4 activity [19].

Although many mRNAs are cleaved by microRNAs, only a subset of these transcripts attract RDR activity and produce endogenous secondary siRNAs (ta-siRNAs) [20]. Presentations by David Baulcombe and James Carrington (Oregon State University, USA) indicate that the critical factor determining whether secondary siRNAs will be produced from a transcript is the size of the miRNA directing transcript cleavage. Most plant miRNAs are 21 nt in length and do not trigger ta-siRNA production, but the small number of miRNAs capable of ta-siRNA initiation exist in a predominant 22 nt form. By modifying the precursor of a 22 nt miRNA so that it instead produced a 21 nt miRNA, ta-siRNA production was abolished without hindering the initial cleavage directed by the miRNA. Likewise, lengthening a 21 nt miRNA by a single base conferred the ability to generate ta-siRNAs. Since both 21 and 22 nt miRNAs are bound by AGO1, James Carrington suggested that a conformational switch (depicted as a "red flash") occurs in the AGO protein, dependent upon miRNA length, to trigger secondary siRNA production. However the RNA bound to AGO proteins might not have a rigid structure and other factors might be involved in sensing the length of bound sRNA.

## Welcome to the Jungle

Our understanding of small RNAs in plants has progressed rapidly since the advent of next-generation deep sequencing technologies and novel categories of small RNAs continue to be uncovered. Yijun Qi (National Institute of Biological Sciences, China) described 24 nt miRNAs in rice. These long miRNAs (lmiRNAs) associate with AGO4-clade Argonaute proteins and trigger asymmetric DNA methylation in *cis *and *trans *but are distinct from siRNAs because they do not require RDR2 for synthesis [21]. Hailing Jin (University of California Riverside, USA) presented evidence of similar 24 nt miRNAs and a 30 nt lmiRNA in *Arabidopsis*. Like their rice counterparts, *Arabidopsis *lmiRNAs are produced from a single strand of an RNA hairpin, require DCL3 and AGO4, and target DNA methylation to homologous DNA. But unlike rice lmiRNAs, *Arabidopsis *lmiRNAs also require RDR2 and Pol IV, which suggests complex biogenesis of these small RNAs in different species.

Xiaofeng Cao (Institute of Genetics and Developmental Biology, China) also described a recently discovered category of small RNA in rice - phased 24 nt siRNAs. In addition to hundreds of loci producing ta-siRNA-like phased 21 nt siRNAs, rice contains many loci producing 24 nt phased siRNAs [22]. These "phasiRNAs" require *DRD701 *(*OsRDR6*) and *OsDCL4*, presumably for production of a double-stranded precursor, but they do not require *OsDCL3a*, the Dicer responsible for cleavage of non-phased 24 nt siRNAs. The increased availability of plant genome sequences and the decreasing price of small RNA deep sequencing will undoubtedly sustain the discovery of novel small RNA classes for some time to come.

## Conclusions

The Keystone Symposium "RNA Silencing Mechanisms in Plants" was a stimulating meeting with many excellent scientific presentations. It is clear that plant systems are poised to unravel many important questions in RNA silencing, including systemic movement and the transgenerational impact of small RNA, interaction between endogenous and virus-derived silencing systems, and mechanics of secondary siRNA production. Solving these riddles and applying plant models to other systems will ensure RNA silencing in plants remains a fertile field.

## Competing interests

The authors declare that they have no competing interests.
